# COU254, a specific 3-carboxamide-coumarin inhibitor of coagulation factor XII, does not protect mice from acute ischemic stroke

**DOI:** 10.1186/2040-7378-2-5

**Published:** 2010-02-15

**Authors:** Peter Kraft, Tobias Schwarz, Lionel Pochet, Guido Stoll, Christoph Kleinschnitz

**Affiliations:** 1 Department of Neurology, Josef-Schneider-Str. 11, University Clinic of Wuerzburg, Germany; 2 Department of Pharmacy, Drug Design and Discovery Center, FUNDP, University of Namur, Belgium

## Abstract

**Background:**

Anticoagulation is an important means to prevent from acute ischemic stroke but is associated with a significant risk of severe hemorrhages. Previous studies have shown that blood coagulation factor XII (FXII)-deficient mice are protected from pathological thrombus formation during cerebral ischemia without bearing an increased bleeding tendency. Hence, pharmacological blockade of FXII might be a promising and safe approach to prevent acute ischemic stroke and possibly other thromboembolic disorders but pharmacological inhibitors selective over FXII are still lacking. In the present study we investigated the efficacy of COU254, a novel nonpeptidic 3-carboxamide-coumarin that selectively blocks FXII activity, on stroke development and post stroke functional outcome in mice.

**Methods:**

C57Bl/6 mice were treated with COU254 (40 mg/kg i.p.) or vehicle and subjected to 60 min transient middle cerebral artery occlusion (tMCAO) using the intraluminal filament method. After 24 h infarct volumes were determined from 2,3,5-Triphenyltetrazoliumchloride(TTC)-stained brain sections and functional scores were assessed. Hematoxylin and eosin (H&E) staining was used to estimate the extent of neuronal cell damage. Thrombus formation within the infarcted brain areas was analyzed by immunoblot.

**Results:**

Infarct volumes and functional outcomes on day 1 after tMCAO did not significantly differ between COU254 pre-treated mice or untreated controls (p > 0.05). Histology revealed extensive ischemic neuronal damage regularly including the cortex and the basal ganglia in both groups. COU254 treatment did not prevent intracerebral fibrin(ogen) formation.

**Conclusions:**

COU254 at the given concentration of 40 mg/kg failed to demonstrate efficacy in acute ischemic stroke in this preliminary study. Further preclinical evaluation of 3-carboxamide-coumarins is needed before the antithrombotic potential of this novel class of FXII inhibitors can be finally judged.

## Introduction

Thromboembolic occlusion of intracerebral vessels is responsible for the majority of ischemic strokes [1]. Studies on the early use of anticoagulant drugs (e.g. heparin) in cerebral ischemia failed to demonstrate overall benefit in that reduced lesion progression was counterbalanced by an increase in hemorrhages [2]. In addition, long-term anticoagulation for secondary prevention of cardioembolic stroke, mainly accomplished by warfarin prescription, is inevitably associated with increased bleeding-related morbidity and mortality [3]. Hence, identification of novel targets for more effective and safer anticoagulation in patients with imminent stroke is badly needed.

In the classical "waterfall model" of blood coagulation the formation of a fibrin thrombus can be initiated by two distinct pathways, the extrinsic and the intrinsic pathway [4]. Both cascades consist of a series of proteolytic reactions involving trypsin-like serine proteases [5]. Fibrin formation via the *extrinsic pathway *occurs when tissue factor (TF), located on cell membranes in the subendothelium of a vessel, forms a complex with activated coagulation factor VII (FVIIa) after endothelial injury [6]. According to the original assumption, the *intrinsic pathway *is initiated when coagulation factor XII (FXII) becomes activated on a negatively charged surface followed by successive activation of factor XI (FXI) and factor IX (FIX) [7]. FXII has long been considered to be dispensable for clot formation because humans with hereditary FXII deficiency suffer from neither spontaneous nor injury-related abnormal bleedings [8,9]. This concept was recently called into question by studies demonstrating that FXII-deficient mice are profoundly protected from pathological thrombus formation in different models of arterial thrombosis but, like FXII-deficient humans, do not show impaired hemostasis [10,11]. Consequently, it was anticipated that the use of FXII inhibitors would be associated with relatively low rates of therapy-related hemorrhages, the major clinical complication associated with current anticoagulant therapies [1]. Indeed, wild-type mice treated with D-Pro-Phe-Arg chloromethyl ketone (PCK), which blocks the amidolytic activity of FXIIa, and subjected to ischemic stroke afterwards, developed less vessel occlusive thrombi in the cerebral microvasculature but did not show increased bleeding tendencies [11]. However, PCK is not selective over FXII and also interacts with other components of the plasmatic coagulation cascade [12].

Pochet and co-workers recently described the synthesis of new 3-carboxamide-coumarins which are the first selective nonpeptidic inhibitors of FXIIa [12]. COU254 is a member of this novel class of FXII inhibitors. In the present study we assessed the effect of COU254 on stroke development, intracerebral fibrinogen clotting and post stroke functional outcome in mice.

## Methods

### Animal experiments

A total of 26 mice were used in this study. Animal experiments were approved by legal state authorities (Bezirksregierung of Unterfranken) and conducted according to the recommendations for research in basic stroke studies [13]. Focal cerebral ischemia was induced in 6-8-weeks old male C57Bl/6 mice (Harlan Winkelmann, Borchen, Germany) by 60 min transient middle cerebral artery occlusion (tMCAO) as described [11,14]. Mice were anesthetized with 2.5% isoflurane (Abbott, Wiesbaden, Germany). Following a midline skin incision in the neck, the proximal common carotid artery and the external carotid artery were ligated and a standardized silicon rubber-coated 6.0 nylon monofilament (6021; Doccol Corp., Redlands, CA, USA) was inserted and advanced via the right internal carotid artery to occlude the origin of the right MCA. The operator was blinded to the treatment groups and operation time per animal did not exceed 15 minutes. The intraluminal suture was left *in situ *for 60 minutes. Then animals were re-anesthetized and the occluding monofilament was withdrawn to allow reperfusion.

COU254 dissolved in 25% DMSO was administered intraperitoneally (i.p.) 2 h before the induction of tMCAO at a dosage of 40 mg/kg bodyweight. Vehicle-treated control mice receiving 25% DMSO without COU254 served as controls. The i.p. route of administration was chosen because i.v. application was afflicted with significant acute toxicity and mortality in preliminary experiments.

### Determination of infarct size and histology

Edema-corrected infarct volumes were quantified by planimetry from 2,3,5-Triphenyltetrazoliumchloride (TTC)-stained brain sections 24 h after ischemic stroke as described [11,14,15]. For morphological assessment, paraffin embedded brains were stained with hematoxylin and eosin (H&E).

### Protein extraction and Western blot analysis

Following TTC staining cortices were dissected from formalin-fixed brain slices and homogenized in RIPA buffer (25 mM Tris pH 7.4, 150 mM NaCl, 1% NP40) containing 2% SDS. The samples were incubated for 20 min at 100°C followed by incubation at 60°C for 2 h [16]. After that, tissue lysates were centrifuged at 15.000 × g for 20 min at 4°C and supernatants were used for BCA protein assay and subsequent Western blot analysis.

The total lysates were treated with SDS-PAGE loading buffer (final conc. 65 mM Tris, 5% 2-mercaptoethanol, 3% SDS, 10% glycerol) at 95°C for 5 min. 30 μg of total protein were electrophoresed and transferred to a PVDF membrane. After blocking for 1 h with blocking buffer (5% nonfat dry milk, 50 mM Tris-HCl pH 7.5, 150 mM NaCl, 0.05% Tween-20) membranes were incubated with the primary antibody at 4°C over night at the following dilutions: anti-Fibrinogen (cross-reactive against fibrin) pAb 1:500 (Acris Antibodies) and anti-Actin mAb 1:10,000 (Dianova). After a washing step with TBS-T (50 mM Tris-HCl pH 7.5, 150 mM NaCl, 0.05% Tween-20), membranes were incubated for 1 h with HRP-conjugated donkey anti-rabbit IgG (for Fibrinogen) or donkey anti-mouse IgG (for Actin) at a dilution of 1:5000 and were finally developed using ECLplus (GE Healthcare).

### Assessment of functional outcome

24 h after tMCAO the modified Bederson score [17] was used to determine global neurological function according to the following scoring system: 0, no deficit; 1, forelimb flexion; 2, decreased resistance to lateral push; 3, unidirectional circling; 4, longitudinal spinning; 5, no movement. Motor function and coordination were evaluated by the grip test [18]. For this test, the mouse was placed midway on a string between two supports and rated as follows: 0, falls off; 1, hangs onto string by one or both forepaws; 2, as for 1, and attempts to climb onto string; 3, hangs onto string by one or both forepaws plus one or both hindpaws; 4, hangs onto string by fore- and hindpaws plus tail wrapped around string; 5, escape (to the supports). Neurological scores were always assessed by an independent and blinded investigator.

### Laser-Doppler flowmetry

Laser-Doppler flowmetry (Moor Instruments, Axminster, U.K.) was used in some animals (n = 3/group) to monitor regional cerebral blood flow (rCBF) in the MCA territory (6 mm lateral and 2 mm posterior from bregma) [19].

### Statistics

Data are expressed as mean ± standard deviation (SD). For statistical analysis, PrismGraph 4.0 software package (La Jolla, CA, USA) was used. Infarct volumes and neurological scores were analyzed using the non-parametric Mann Whitney test. Laser Doppler flowmetry data were compared by 1-way ANOVA followed by Bonferroni post hoc test. P-values < 0.05 were considered to be statistically significant.

## Results

**Figure 1:**
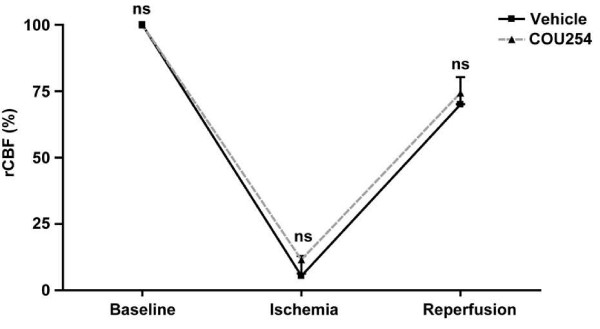
**rCBF does not differ between COU254-treated mice and controls after tMCAO**. Determination of regional cerebral blood flow (rCBF) using Laser Doppler flowmetry before the occlusion of the middle cerebral artery (baseline), 10 min after the occlusion (ischemia) and again 10 min after the removal of the filament (reperfusion) in COU254-treated mice and vehicle-treated controls (n = 3/group). No significant differences in rCBF were observed between the two groups. 1-way ANOVA, Bonferroni post hoc test, ns = not significant.

The transient middle cerebral artery filament occlusion model (tMCAO) was used to induce focal cerebral ischemia in mice [11,14,15]. After advancing the filament to the origin of the MCA the decrease in rCBF was similar between control mice and COU254-treated mice (5.4 ± 7.5% of baseline levels vs. 11.5 ± 5.2% of baseline levels; p > 0.05) (Figure 1). Ten minutes after reperfusion rCBF in the MCA territory was reconstituted to >70% of baseline levels and again did not significantly differ between treated and untreated mice (70.0 ± 10.3% of baseline levels vs. 74.3 ± 4.2% of baseline levels; p > 0.05) (Figure 1). Taken together, these findings exclude any significant rCBF alterations related to COU254 or vehicle treatment and prove that MCA occlusion and reperfusion were sufficient in our model.

**Figure 2:**
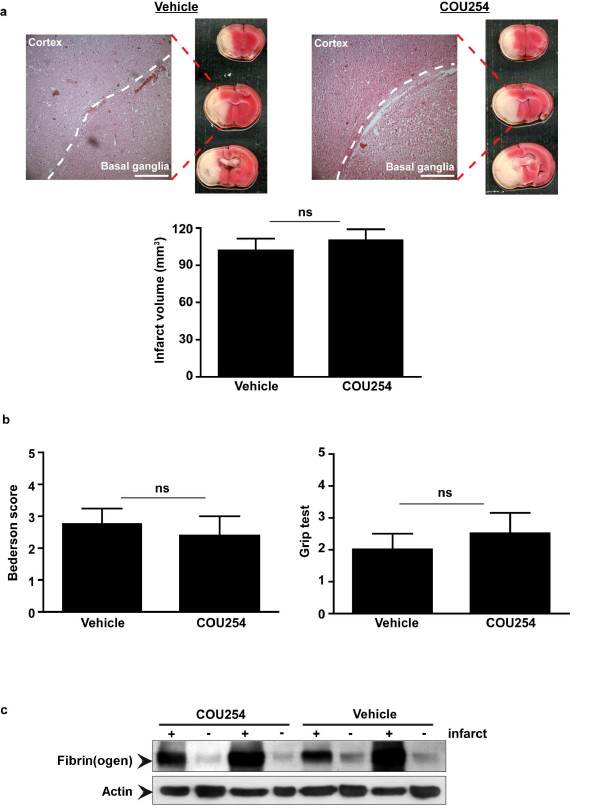
**COU254 does not improve outcome after experimental stroke in mice**. Infarct size and functional outcome in COU254-treated mice and controls (vehicle) 24 h after 60 min transient middle cerebral artery occlusion (tMCAO). (**a**) (top) Representative 2,3,5-Triphenyltetrazoliumchloride (TTC)-stained coronal brain sections from the two animal groups. Ischemic infarctions appear white and regularly include the neocortex and basal ganglia as confirmed by hematoxylin and eosin (H&E) staining (bar represents 250 μm). (bottom) Infarct volumes on day 1 after tMCAO in COU254-treated mice and vehicle-treated controls as determined by planimetry (n = 10/group). Non-parametric Mann Whitney test, ns = not significant. (**b**) Neurological Bederson score and grip test score on day 1 after tMCAO in COU254-treated mice and vehicle-treated controls. In line with the results on infarct volumes, no significant functional differences became apparent between the treatment groups. Non-parametric Mann Whitney test, ns = not significant. (**c**) Accumulation of fibrin(ogen) in the infarcted (+) and contralateral (-) cortices of COU254-treated mice or vehicle-treated controls. Fibrinogen clotting 24 h after ischemia was analyzed by immunoblotting. Two representative immunoblots of each group are shown.

As a next step, we determined infarct sizes and the extent of neuronal damage in control mice and mice treated with COU254. 24 h after tMCAO no significant differences in infarct volumes were observed between the two groups as revealed by 2,3,5-Triphenyltetrazoliumchloride (TTC) staining and successive infarct planimetry (101.5 ± 31.4 mm^3 ^vs. 110.0 ± 27.2 mm^3^; p > 0.05) (Figure 2a). In line with these findings, H&E staining confirmed widespread ischemic neurodegeneration in both groups which regularly expanded to the basal ganglia and the neocortex (Figure 2a). Detailed analysis of the neurological status using two different functional scores also could not reveal any beneficial effects of COU254 in acute ischemic stroke (Bederson score: 2.8 ± 1.4 vs. 2.4 ± 1.8; p > 0.05; grip test: 2.0 ± 1.4 vs. 2.5 ± 1.9; p > 0.05) (Figure 2b). Finally, no differences in thrombus formation within the infarcted brain areas of COU254-treated mice or controls were detectable by immunoblot (Figure 2c).

## Discussion

Unexpectedly, the selective nonpeptidic FXIIa inhibitor COU254 could not ameliorate ischemic brain damage after tMCAO in mice.

COU254 belongs to a new group of recently described 3-carboxamide-coumarins which represent the first selective inhibitors of FXIIa [12]. In contrast to conventional FXII inhibitors it does not interfere with other components of the contact activation system or plasmatic coagulation cascade potentially involved in stroke development such as kallikrein, FXa or the TF/FVIIa complex [15]. Mechanistically, COU254 mediates anti-FXII activity through the formation of an acyl enzyme instead of an alkyl enzyme as observed with thrombin and 6-chloromethyl ester coumarins [12]. Several reports have meanwhile highlighted the role of FXII in pathological thrombogenesis [20]. FXII-deficient mice were protected against arterial thrombosis, collagen-induced venous thromboembolism and ischemic stroke [10,11]. Importantly, *FXII*^-/- ^mice display normal hemostasis and consequently FXIIa inhibition was not associated with increased bleeding complications supporting the intriguing hypothesis that hemostasis and thrombosis are two mechanistically different processes [20]. Hence, FXIIa is considered an attractive target for pharmacological inhibitors designed to treat or prevent thromboembolic disorders. Such a safe therapy might be particularly advantageous for the treatment of acute ischemic stroke, where the conventional anticoagulants used against stroke progression or recurrence are inheritably associated with increased bleeding-related morbidity and mortality [1].

We recently established D-Pro-Phe-Arg chloromethyl ketone (PCK) as FXIIa inhibitor in experimental stroke [11]. Mice treated with PCK immediately before the occlusion of the middle cerebral artery (MCA) developed smaller infarcts and less severe neurological deficits compared to controls. Moreover, the formation of fibrin within the infarcted brains was significantly reduced. Although PCK irreversibly inhibits the amidolytic activity of FXIIa it is not specific over FXII. Rather, PCK has been shown to block other components of the contact activation system or plasmatic coagulation cascade bearing the potential risk of undesired adverse effects [12]. Moreover, the peptidic structure and the alkylating behavior of the chloromethyl function prevent the application of PCK as oral anticoagulant. Natural anticoagulant proteins displaying anti-FXIIa activity were also reported, e.g. from leguminous plants [21], hematophagous insects [22-24], helminth parasites [25] and bacteria [26]. Again, despite their proven efficacy, all these proteins were generally not selective over blood coagulation proteases.

Several reasons might account for the negative results in present study. Besides "true" inefficacy of COU254 in acute experimental stroke related for example to the relatively low FXIIa inhibitory potency of COU254 compared to PCK [12] technical limitations could have been responsible. The anti-FXIIa activity of COU254 has only been established from in vitro dose-response curves so far and pharmacodynamic or pharmacokinetic data on COU254 in animals, especially rodents, are lacking. Moreover, the optimum dosage or route of application of COU254 in mice is yet unknown as is the ideal time point of administration during the course of ischemic stroke. Because human and mouse FXII share a high degree of sequence homology and the established human FXII inhibitors usually also block murine FXII [10,11], species specific differences of COU254 mode of action between humans and rodents seem unlikely but cannot be completely ruled out.

In summary, 3-carboxamide-coumarins represent a promising new class of selective FXII inhibitors but further preclinical evaluation of these compounds in animal models is clearly needed before any firm conclusions on their antithrombotic potential can be drawn.

## Competing interests

The authors declare that they have no competing interests.

## Authors' contributions

All authors have read and approved the final manuscript.

PK operated the animals, assessed the functional scores and interpreted the data. TS performed the immunoblots. LP provided COU254 and finalized the manuscript. GS and CK conceived the experiments, funded the project and wrote the manuscript.
